# Frequency of Hospitalized Infections Is Reduced in Rheumatoid Arthritis Patients Who Received Biological and Targeted Synthetic Disease-Modifying Antirheumatic Drugs after 2010

**DOI:** 10.1155/2018/6259010

**Published:** 2018-08-14

**Authors:** Kunihiro Ichinose, Toshimasa Shimizu, Masataka Umeda, Shoichi Fukui, Ayako Nishino, Tomohiro Koga, Shin-ya Kawashiri, Naoki Iwamoto, Mami Tamai, Hideki Nakamura, Shuntaro Sato, Tomoki Origuchi, Atsushi Kawakami

**Affiliations:** ^1^Department of Immunology and Rheumatology, Unit of Advanced Preventive Medical Sciences Division of Advanced Preventive Medical Sciences, Nagasaki University Graduate School of Biomedical Sciences, Nagasaki, Japan; ^2^Clinical Research Center, Nagasaki University Hospital, Nagasaki, Japan; ^3^Rehabilitation Sciences, Nagasaki University Graduate School of Biomedical Sciences, Nagasaki, Japan

## Abstract

**Background:**

Biological disease-modifying antirheumatic drugs (bDMARDs) and targeted synthetic (ts) DMARDs are important in rheumatoid arthritis (RA) treatment. The risk of hospitalized infection associated with bDMARDs/tsDMARDs in RA patients is unclear.

**Methods:**

We retrospectively analyzed the cases of the 275 RA patients with 449 treatment episodes who were administered a bDMARD/tsDMARD at Nagasaki University Hospital in July 2003–January 2015. We determined the incidence and risk factors of infection requiring hospitalization in the patients during a 3-year observation period.

**Results:**

Thirty-five (12.7%) of the patients experienced a hospitalized infection. The hospitalized infection risk did not differ significantly among several bDMARDs/tsDMARDs. A multivariate analysis revealed that the comorbidities of chronic lung disease (adjusted HR 5.342, 95% CI 2.409–12.42, *p* < 0.0001) and the initiation of bDMARDs/tsDMARDs before 2010 (adjusted HR 4.266, 95% CI 1.827–10.60, *p* = 0.0007) are significant independent risk factors for hospitalized infection. Compared to the before-2010 group, the group of patients whose treatment initiated in 2010 or later showed higher patient ages at the initiation of bDMARD/tsDMARD treatment and a higher rate of the use of prophylaxis with an antituberculosis agent, whereas the disease activities and number of the patients who received >5 mg of prednisolone were lower in the after-2010 group.

**Conclusions:**

This is the first report that the frequency of hospitalized infection significantly decreased when the patients were treated with a bDMARD or tsDMARD after 2010. Our results indicate that the updated announcement of diagnosis and treatment criteria might contribute to a reduced risk of hospitalized infection and a better understanding of the use of bDMARDs/tsDMARDs by rheumatologists.

## 1. Introduction

Rheumatoid arthritis (RA) is a systemic inflammatory disease with arthritis that is induced by an autoimmune mechanism. In the last decade, there have been significant advances in the treatment of RA, especially for patients whose arthritis does not respond to conventional disease-modifying antirheumatic drugs (DMARDs). Among the DMARDs, biological DMARDs (bDMARDs) and targeted synthetic (ts) DMARDs are the mainstay treatment for RA. Biologic agents commonly used in Japan for the treatment of RA include antitumor necrosis factor (anti-TNF) agents such as infliximab (IFX), etanercept (ETA), adalimumab (ADA), golimumab (GLM), and certolizumab pegol (CZP); an inhibitor of T-cell costimulation (abatacept (ABT)); an inhibitor of interleukin- (IL-) 6 receptor (tocilizumab (TCZ)); and a small-molecule Janus-associated kinase (Jak) inhibitor (tofacitinib (Tofa) and baricitinib). In recent years, it has become possible to control RA by treatment with bDMARDs/tsDMARDs, but the immunosuppression induced by bDMARD/tsDMARD treatment has led to problems with hospitalized infections. Not only opportunistic infections such as *Pneumocystis jiroveci* pneumonia (PCP), tuberculosis, and nontuberculous mycobacteriosis (NTM) have been observed in compromised hosts; general bacterial pneumonia and herpes zoster are also seen.

Although it is still controversial regarding whether bDMARDs/tsDMARDs increase the risk of infectious diseases [1, 2], a study of side effects in RA patients treated with TNF inhibitors in Japan showed that their risk of serious infection was higher compared to the non-TNF inhibitor treatment group (odds ratio 2.37; 95% confidence interval (CI) 1.11–5.05) [3]. There is also a report that RA itself raises the risk of infection with immune abnormality [4], but the underlying mechanism of this has not been clarified.

The 2010 American College of Rheumatology (ACR)/European League Against Rheumatism (EULAR) classification criteria focus on features that would identify patients at an earlier stage of disease compared to the previously used criteria that had been last revised in 1987 [5–7]. In an early-RA cohort study, the sensitivity to start the treatment with a DMARD or methotrexate (MTX) was 74%–84% in the 2010 criteria, but 47%–68% in the 1987 ACR classification [8–10]. After the 2010 criteria were published, RA patients at earlier stages were identified and treated promptly.

In this study, we examined the risk of hospitalized infection associated with bDMARDs/tsDMARDs in RA patients. Regarding the definition of hospitalized infections, we referred to a previous report [11] describing “fatal case,” “hospitalization,” and “administration of intravenous antibiotics” as criteria.

## 2. Methods

### 2.1. Patients and Methods

For the period from July 2003 to January 2015, 275 RA patients with 449 treatment episodes were treated with IFX, ETA, ADA, CZP, GLM, ABT, TCZ, or Tofa at Nagasaki University Hospital. All of these patients fulfilled the 1987 criteria or the 2010 criteria of RA [5, 6]. A treatment episode was defined as the initiation of a new course of bDMARD/tsDMARD.

We retrospectively analyzed the incidence and risk factors of infection requiring hospitalization in the RA patients using bDMARDs/tsDMARDs during a 3-year observation period. An outcome was the first occurrence of hospitalized infection during the 3-year observation periods. The start date was defined as the point of diagnosis. Follow-up ended at the earliest of the following: a first hospitalization that resulted from infection, reaching the end of the 3-year period after the initiation of the bDMARD/tsDMARD, the time that a currently used bDMARD/tsDMARD was discontinued, loss to follow-up, and death. We categorized the types of hospitalized infections as pneumonia and respiratory tract, skin and soft tissue, genitourinary tract, sepsis/bacteremia, or other [12].

The number of months of exposure to each biological agent was assigned as the number of months between the index date and the endpoint date. If the drug was discontinued, the patient was considered to have been exposed to that specific biological agent until the end of the recommended dosing interval. The observation of the patients who were switched to a new course of treatment with a different bDMARD or tsDMARD was continued until the follow-up ended. The study was reviewed and approved by the Medical Ethics Committee of Nagasaki University Hospital (approval number 05072959).

## 3. Data Collection

We retrospectively reviewed each patient's medical records. Demographic data, clinical information, and laboratory information including RA-related features were collected. Comorbid diseases such as diabetes mellitus and chronic lung disease were also examined at the baseline. Diabetes was defined as fasting blood glucose level ≥ 126 mg/dl, serum HbAc1 ≥ 6.0%, or the use of a diabetes medication. Comorbidities of lung disease included interstitial lung disease, bronchiolitis, bronchiectasis, and pulmonary emphysema, which were diagnosed according to abnormal findings on high-resolution computed tomography (HRCT). The patients who received MTX, prednisolone (PSL), or prophylaxis with an antituberculosis agent or trimethoprim-sulfamethoxazole (TMP-SMX) at the induction of the bDMARD/tsDMARD were also examined.

### 3.1. Statistical Analyses

A nonparametric Wilcoxon rank-sum test was used for intergroup comparisons of multiple variables. Fisher's exact test was also performed to test the possible association between each variable factor and the divided groups. We performed univariate and multivariable competing risk regression analyses to determine the predictive factors of hospitalized infections. The data of time to the hospitalized infection were analyzed using the Kaplan-Meier method with a log-rank test. All of the statistical analyses were performed using JMP® Pro 13 (SAS Institute, Cary, NC, USA). The significance level was set at *p* < 0.05.

## 4. Results

### 4.1. Patient Characteristics

The median duration of follow-up was 49.8 months. Thirty-five (12.7%) of the 275 patients experienced a hospitalized infection. The characteristics of the patients are summarized in Table 1. The infection group had a significantly higher percentage of males (*p* = 0.0197) and significantly older age at the start of bDMARDs/tsDMARDs (*p* = 0.0325); these patients were also significantly more likely to be administered prophylaxis with an antituberculosis agent (*p* = 0.0458) or TMP-SMX (*p* = 0.0290), and the group had a significantly higher rate of comorbidities of chronic lung disease (*p* < 0.0001).

**Figure 1:**
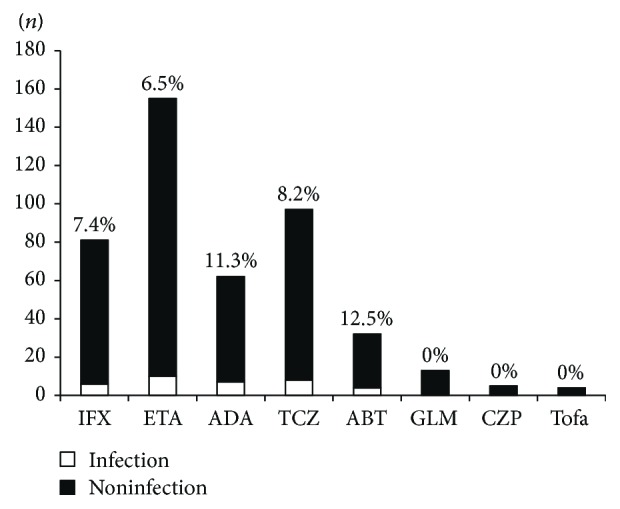
The number and percentage of hospitalized infections during the treatment with each biological disease-modifying antirheumatic drug (bDMARD)/targeted synthetic disease-modifying antirheumatic drug (tsDMARD). Open bars: the hospitalized infection group. Closed bars: the noninfection group. IFX: infliximab; ETA: etanercept; ADA: adalimumab; TCZ: tocilizumab; ABT: abatacept; GLM: golimumab; CZP: certolizumab pegol; Tofa: tofacitinib.

There were 449 treatment episodes with bDMARDs/tsDMARDs. Among these episodes, 81 (18.0%) were with IFX, 155 (34.5%) were with ETA, 62 (13.8%) were with ADA, 97 (21.6%) were with TCZ, 32 (7.1%) were with ABT, 13 (2.9%) were with GLM, five (1.1%) were with CZP, and four (0.9%) were with Tofa. The number and percentage of hospitalized infections (*n* = 35) during the treatment with each bDMARD/tsDMARD were as follows: six (7.4%) with IFX, 10 (6.5%) with ETA, seven (11.3%) with ADA, eight (8.2%) with TCZ, and four (12.5%) with ABT. There were no hospitalized infections in the patients who received GLM, CZP, and Tofa (Figure 1). The incidence of hospitalized infections during the treatment with each bDMARD/tsDMARD in the 35 patients with hospitalized infections was as follows: ten (29%) with ETA, eight (23%) with TCZ, seven (20%) with ADA, six (17%) with IFX, and four (11%) with ABT.

### 4.2. Incidence of Hospitalized Infection by Infection Sites

The type and number of hospitalized infections across treatment groups during the follow-up are shown in Table 2. Respiratory infection (64.9%) (especially bacterial pneumonia) occurred most frequently in the patients who incurred a hospitalized infection, and PCP (10.5%), tuberculosis (7.9%), NTM (7.9%), acute exacerbation of chronic lower respiratory disease (5.3%), and fungus infection (5.3%) followed in frequency. Other than respiratory infection, herpes zoster (10.5%) was most frequently seen. Among the 35 patients who developed a hospitalized infection, four (11.4%) died during the follow-up.

### 4.3. Odds Ratio for Hospitalized Infection according to Different bDMARDs/tsDMARDs

**Figure 2:**
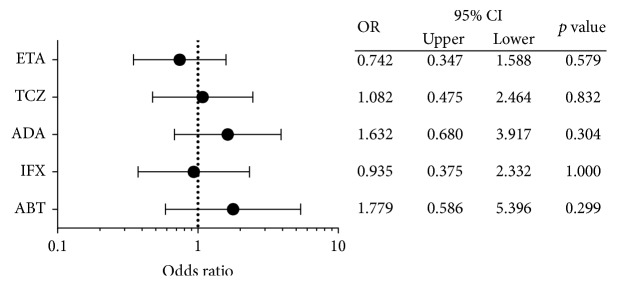
Odds ratio for hospitalized infection according to different biological disease-modifying antirheumatic drug. ETA: etanercept; TCZ: tocilizumab; ADA: adalimumab; IFX: infliximab; ABT: abatacept.

The risk of hospitalized infection across different bDMARDs in the 449 treatment episodes is shown in Figure 2. Our analysis revealed no significant differences among the treatments with ETA, TCZ, ADA, IFX, and ABT in this cohort. The comparison of the TNF inhibitors (IFX, ETA, ADA, GLM, and CZP) with the non-TNF inhibitors (TCZ and ADA) revealed that the frequency of hospitalized infection was 23 of 316 patients (7.3%) and 12 of 129 patients (9.3%), respectively, with no significant difference (*p* = 0.5604).

### 4.4. Incidence of Hospitalized Infection in the Patients Who Started bDMARD Treatment before versus from 2010 Onward

**Figure 3:**
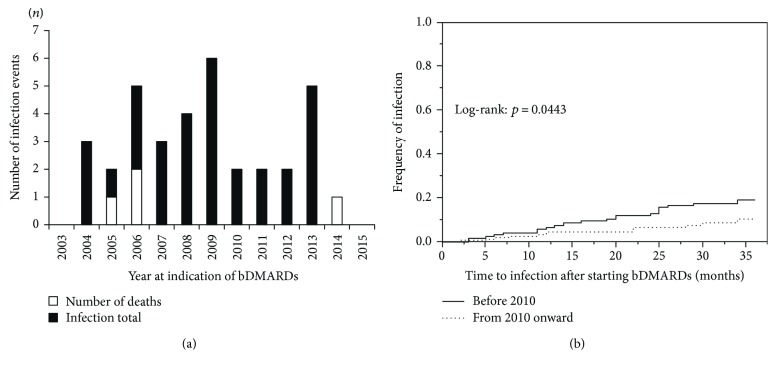
(a) Number of infections in the patients for whom a biological disease-modifying antirheumatic drug (bDMARD) was initiated each year. Open bars: the number of deaths in each year due to infections. Closed bars: the total number of infections for each year. (b) Cumulative incidence of hospitalized infections during the 3-year follow up, according to bDMARD.

We next analyzed the numbers of infections in the patients who started bDMARD treatment each year from 2003 to 2015 (Figure 3(a)). In the univariate model, 23 (65.7%) of the 35 patients in the infection group and 100 (41.7%) of the 240 patients in the noninfection group were first administered a bDMARD in the years before 2010, and these percentages are significantly different (*p* = 0.0102) (Table 1). In our comparison of the incidence of infections by year of bDMARD introduction during the 3 years after starting bDMARDs (with the Kaplan-Meier method), the log-rank test showed that the incidence of hospitalized infections was significantly increased in the patients for whom a bDMARD was introduced before 2010 (*p* = 0.0443) (Figure 3(b)).

### 4.5. Stepwise Logistic Regression

The predictors of hospitalized infection in the stepwise logistic regression analyses are shown in Table 3. In the multivariable model, gender, age at starting a bDMARD/tsDMARD, number of previous bDMARD/tsDMARD uses, prophylaxis with an antituberculosis agent, comorbidities of chronic lung disease, and initiation of a bDMARD/tsDMARD before 2010 were selected (*p* < 0.05) from the univariate model. In the stepwise logistic regression model, the independent predictors of hospitalized infection were comorbidities of chronic lung disease (adjusted hazard ratio (HR) 5.342, 95% CI 2.409–12.42, *p* < 0.0001) and initiation of bDMARD/tsDMARD before 2010 (adjusted HR 4.266, 95% CI 1.827–10.60, *p* = 0.0007).

### 4.6. The Backgrounds of the Patients Whose bDMARD/tsDMARD Treatment Was Initiated before 2010 versus from 2010 Onward

Recent changes in the concept of treatment for RA may contribute to the lower risk of hospitalized infection that we observed in the present after-2010 group. The backgrounds of the patients whose bDMARD/tsDMARD treatment was initiated before 2010 versus from 2010 onward are shown in Table 4. Compared to the before-2010 group, the group of patients whose treatment initiated in 2010 or later showed higher patient ages at the initiation of bDMARD/tsDMARD treatment (*p* = 0.0185) and a higher rate of the use of prophylaxis with an antituberculosis agent (*p* = 0.0163), whereas the DAS28-CRP (*p* = 0.0003), DAS28-ESR (*p* = 0.0026), and number of the patients who received >5 mg of PSL (*p* < 0.0001) were lower in the after-2010 group.

## 5. Discussion

This retrospective study provides the first report that the rate of hospitalized infections among RA patients that occurred within 3 years after the initiation of treatment with a bDMARD/tsDMARD decreased significantly in the patients whose bDMARD or tsDMARD treatment began after 2010. We also observed that there was no significant difference in the rate of hospitalized infections among the different bDMARDs during the 3-year follow-up. In the stepwise logistic regression models, the patients who experienced a hospitalized infection were significantly more likely to have the comorbidity of chronic lung disease and the initiation of bDMARD/tsDMARD treatment before 2010. Moreover, respiratory infection was the most frequent infection requiring hospitalization during bDMARD/tsDMARD therapy. Several studies with meta-analysis data compared the risk of serious infection among different biologic agents in randomized controlled trials (RCTs) and open-label extension studies [1, 13–17]. The authors of those studies concluded that some biological agents might contribute to an increase in the risk of serious infection compared to others [1, 17], but other studies showed no significant association with the risk of serious infection during biological therapies at the recommended doses [13–16, 18]. Although in the present study the number of patients who were treated with newly approved bDMARDs/tsDMARDs such as GLM, CZP, and Tofa might not be high enough for a conclusion to be made in this regard, the results concerning the comparative risk of hospitalized infection related to bDMARDs/tsDMARDs are somewhat diverse across studies. Thus, we agree with the idea that a broader variability in patients' risk of hospitalized infection is related to demographics, comorbidities, higher prednisolone doses, and other patient-specific risk factors compared to exposure to a bDMARD/tsDMARD [19].

In the univariate model **(** Table 1 **)**, the increased incidence of hospitalization due to infection in those patients on bDMARDs and tsDMARDs receiving either prophylaxis with antituberculosis agents (*p* = 0.0458) or TMP-SMX (*p* = 0.0290). The age at initiation of bDMARD/tsDMARD was significantly higher in those who received antituberculosis agents (*p* < 0.001) and TMP-SMX (*p* = 0.0110). There is undoubtedly an influence of older age on the infection risk in RA [20]. Thus, frequency of hospitalized infection was thought to be higher in the group who received these prophylaxises.

Several reports showed that comorbidities of chronic lung disease are predictive of hospitalized infections in Japan [3, 21, 22]. As in our study, respiratory infection (especially bacterial pneumonia) is the most commonly seen in all infections [21]. The patients' backgrounds based on the presence/absence of chronic lung disease in our study are summarized in Supplementary Table 1. Male gender (*p* = 0.0186), older age at the initiation of bDMARD/tsDMARD treatment (*p* = 0.0025), higher disease activity (DAS28-CRP; *p* = 0.0237, DAS28-ESR; *p* = 0.0397), and the coexistence of diabetes mellitus (*p* = 0.0245) were seen more often in the presence of chronic lung disease compared to the absence of chronic lung disease. These results are consistent with previous reports [23]. A study of 149 RA patients (mean age 68.0 years; 68 men, 81 women) with pulmonary infections revealed the coexistence of chronic airway lesions with *Pseudomonas aeruginosa* in all exacerbations of bronchiectasis [24]. It has also been shown that chronic lung diseases including chronic obstructive pulmonary disease and idiopathic interstitial pneumonia were associated with colonization by *Pneumocystis jirovecii* in a general population [25, 26], and the same reason may be applied to the incidence of PCP in RA patients.

A recent nationwide database study in Japan showed that the age at the onset of RA has increased over the last decade [27]. Despite these circumstances, the disease activity of RA is much improved. The ACR/EULAR 2010 classification criteria for RA [5, 6], the ACR/EULAR 2010, 2013, and 2016 recommendations for the management of RA [28], and the updated guidelines for TNF inhibitors by the Japan College of Rheumatology (JCR) in 2010, 2012, 2014, 2015, and 2017 [29], for tocilizumab in 2010, 2012, 2013, 2014, and 2017 [30], and for abatacept in 2010, 2014, and 2017 [31] have recommended that rheumatologists begin administering treatment at an earlier stage of RA, and these guidelines have also publicized the known evidence of the risk of hospitalized infection, including higher-dose steroid usage, higher disease activity, and comorbidities of chronic lung disease, which are consistent with our findings. In the present study, although the difference was not significant, the disease duration until bDMARD/tsDMARD initiation was shorter in the after-2010 group compared to the before-2010 group (median: 43 months versus 67.5 months) (*p* = 0.1632) **(** Table 4 **)**, indicating that rheumatologists have been initiating bDMARD/tsDMARD treatment of their RA patients earlier in the course of disease, in order to obtain better outcomes.

The limitations of our study deserve some discussion. First, our population was a relatively small number of patients treated at a single center. We also could not evaluate the relative risk of hospitalized infection in the newly approved bDMARDs/tsDMARDs. Multicenter studies with larger numbers of patients are needed to establish evidence. Second, this study was performed retrospectively, which may confer certain inherent limitations on the results. A selection bias of the enrolled patients might have occurred based on clinical reasons, including the known infections, respiratory dysfunction, and poor performance status, for which bDMARDs/tsDMARDs are generally not chosen for patients with risk factors. Third, information about smoking status, comorbidities such as cardiac disorders, and history of previous infections was not available. For the most precise evaluation of the effects of using bDMARDs/tsDMARDs on the risk of hospitalized infection, both these factors and the duration of administration and doses of bDMARDs/tsDMARDs should be included in the analysis.

In conclusion, our present findings comprise the first report that the risk of hospitalized infection occurring within 3 years after the initiation of bDMARDS/tsDMARDs was significantly decreased when the bDMARDs/tsDMARDs were started after 2010. We also observed that there was no significant difference in the incidence of hospitalized infections among the different bDMARDs during the 3-year observation. The patients who experienced a hospitalized infection were significantly more likely to have comorbidities of chronic lung disease and the initiation of bDMARDs/tsDMARDs before 2010. Also, compared to the before-2010 group, the group of patients whose treatment initiated in 2010 or later showed higher patient ages at the initiation of bDMARD/tsDMARD treatment and a higher rate of the use of prophylaxis with an antituberculosis agent, whereas the DAS28-CRP, DAS28-ESR, and number of the patients who received >5 mg of PSL were lower in the after-2010 group. Our results indicate that the updated announcement of diagnosis and treatment criteria might contribute to the reduction in the risk of hospitalized infection and a better understanding of the use of bDMARDs/tsDMARDs by rheumatologists.

## Figures and Tables

**Table 1:** Characteristics of the total, infection, and noninfection patient groups.

Variable	Total	Infection	Noninfection	*p* value
	(*n* = 275)	(*n* = 35)	(*n* = 240)	
Gender, male (%)	52/275 (18.9%)	12/35 (34.3%)	40/240 (16.7%)	0.0197^∗^
Age at initiation of bDMARD/tsDMARD (IQR)	59 (48–68)	61 (46–67)	58.5 (56–69)	0.0325^∗^
Disease duration until bDMARD/tsDMARD induction (months)	49.5 (14–150)	61 (20–173)	48 (14–149)	0.4677
DAS28-CRP (IQR)	4.60 (3.74–5.46)	4.95 (4.05-6.09)	4.54 (3.69–5.40)	0.1061
DAS28-ESR (IQR)	5.32 (4.37–6.18)	5.69 (4.54–6.74)	5.20 (4.34–6.08)	0.0805
Body weight (kg) (IQR)	52 (46–59)	54 (45–62)	52 (47–58)	0.7788
No. of previous bDMARD/tsDMARD uses (IQR)	1 (1-2)	1 (1-2)	1 (1-2)	0.226
Prophylaxis with an antituberculosis agent	195/275 (70.9%)	30/35 (85.7%)	165/240 (68.8%)	0.0458^∗^
Prophylaxis with TMP-SMX	6/275 (2.18%)	3/35 (8.6%)	3/240 (1.25%)	0.0290^∗^
Comorbidities of chronic lung disease	83/275 (30.2%)	22/35 (62.9%)	61/240 (25.5%)	<0.0001^∗^
Number of PSL ≥5 mg users	115/275 (41.8%)	15/35 (42.9%)	100/240 (41.7%)	1
MTX use	163/275 (59.3%)	19/35 (54.3%)	144/240 (60.0%)	0.582
Diabetes mellitus (%)	33/275 (33%)	7/35 (20.0%)	26/240 (10.8%)	0.1582
Initiation of bDMARD/tsDMARD before 2010	123/275 (44.7%)	23/35 (65.7%)	100/240 (41.7%)	0.0102^∗^

DAS28: disease activity score including 28-joint count; CRP: C-reactive protein; bDMARD: biological disease-modifying antirheumatic drug; tsDMARDs: targeted synthetic disease-modifying antirheumatic drug; MTX: methotrexate; TMP-SMX: trimethoprim-sulfamethoxazole. Values are median (interquartile (IQR)). The *p* values were determined using the nonparametric Wilcoxon signed-rank test and Fisher's exact test. ^∗^ *p* < 0.05.

**Table 2:** Classification of infection and its frequency of infection and whole group.

Type of infection	*n* (% of infection/% of total)
Respiratory infection:	24 (64.9%/8.72%)
Bacterial pneumonia	10 (26.3%/3.64%)
*Pneumocystis jiroveci* pneumonia	4 (10.5%/1.45%)
Tuberculosis	3 (7.9%/1.1%)
Nontuberculous mycobacteria	3 (7.9%/1.1%)
Acute exacerbation of chronic lower respiratory diseases	2 (5.3%/0.72%)
Fungus infection	2 (5.3%/0.72%)
Herpes zoster	4 (10.5%/1.45%)
Skin/soft tissue	2 (5.4%/0.73%)
Sepsis	2 (5.4%/0.73%)
Acute sinusitis	1 (2.7%/0.36%)
Kidney/urinary	1 (2.7%/0.36%)
Bone/joint	1 (2.7%/0.36%)
Digestive	1 (2.7%/0.36%)
Viral infection	1 (2.7%/0.36%)

**Table 3:** The risk of infection in bDMARD uses analyzed with stepwise logistic regression.

Variable	Stepwise logistic regression	
	Adjusted HR (95% CI)	*p* value
Gender, male (%)	1.648 (0.669–3.897)	0.2705
Age at initiation of bDMARD/tsDMARD	1.033 (1.001–1.070)	0.0526
Number of previous bDMARD/tsDMARD uses	1.259 (0.797–1.931)	0.3137
Prophylaxis with an antituberculosis agent	2.502 (0.909–8.200)	0.0775
Comorbidities of chronic lung disease	5.342 (2.409–12.42)	<0.0001^∗^
Initiation of bDMARD/tsDMARD before 2010	4.266 (1.827–0.60)	0.0007^∗^

bDMARD: biological disease-modifying antirheumatic drug; tsDMARDs: targeted synthetic disease-modifying antirheumatic drug. ^∗^ *p* < 0.05.

**Table 4:** Background of the patients with bDMARD/tsDMARD initiation before 2010 and from 2010 to 2015.

Variable	Before 2010 (*n* = 123)	from 2010 to 2015 (*n* = 152)	*p* value
Gender, male (%)	24/123 (19.5%)	28/152 (18.42%)	0.8774
Age at starting bDMARD/tsDMARD (IQR)	57 (46–65)	61 (49–70)	0.0185^∗^
Disease duration until bDMARD/tsDMARD initiation (months)	67.5 (16.5–162.8)	43 (13–148.8)	0.1632
DAS28-CRP (IQR)	4.83 (4.18–6.01)	4.38 (3.46–5.18)	0.0003^∗^
DAS28-ESR (IQR)	5.70 (4.56–6.53)	5.04 (4.21–5.99)	0.0026^∗^
Body weight (kg) (IQR)	51.5 (45.2–56.9)	53.0 (47.6–61.4)	0.0738
Number of previous bDMARD/tsDMARD uses (IQR)	1.5 (1-2)	1 (1–2)	0.0017^∗^
Prophylaxis with an antituberculosis agent	78/123 (63.4%)	117/152 (77.0%)	0.0163^∗^
Prophylaxis with TMP-SMX	4/123 (3.25%)	2/152 (1.32%)	0.4126
Number of PSL ≥5 mg users	70/123 (56.9%)	45/152 (29.6%)	<0.0001^∗^
MTX use	76/123 (61.8%)	87/152 (57.2%)	0.1866
Complication of chronic lung disease	33/123 (26.8%)	50/152 (32.9%)	0.2931
Diabetes mellitus (%)	14/123 (11.4%)	19/152 (12.5%)	0.853

DAS28: disease activity score including 28-joint count; CRP: C-reactive protein; bDMARD: biological disease-modifying antirheumatic drug; tsDMARDs: targeted synthetic disease-modifying antirheumatic drug; MTX: methotrexate; TMP-SMX: trimethoprim-sulfamethoxazole. Values are median (interquartile (IQR)). The *p* values were determined using the nonparametric Wilcoxon signed-rank test and Fisher's exact test. ^∗^ *p* < 0.05.

## Data Availability

The data used to support the findings of this study are available from the corresponding author upon request.

## Supplementary Materials

Supplementary Materials Supplementary Table 1: patient background by presence or absence of chronic lung disease.
