# Supplementary material for: Frequency of Hospitalized Infections Is Reduced in Rheumatoid Arthritis Patients Who Received Biological and Targeted Synthetic Disease-Modifying Antirheumatic Drugs after 2010
Source: J Immunol Res. 2018 Aug 14;2018:6259010. doi: 10.1155/2018/6259010 (PMC6112083; doi:10.1155/2018/6259010)
Supplement: Supplementary Materials — Supplementary Table 1: patient background by presence or absence of chronic lung disease. [file 6259010.f1.pdf]

## “Supplementary Material”

**Supplementary Table 1.** Patient background by presence or absence of chronic lung disease

**Supplementary Table 1.** Patient background by presence or absence of chronic lung disease

| Variable | With chronic lung disease (n=83) | Without chronic lung disease (n=192) | *p*-value |
| --- | --- | --- | --- |
| Gender, male (%) | 23/83 (27.7%) | 29/192 (15.1%) | 0.0186* |
| Age at starting bDMARD/tsDMARD (IQR) | 63 (55–70) | 57 (46–65) | 0.0025* |
| Disease duration until bDMARD/tsDMARD initiation (month) | 65.5 (20.8–176.8) | 47 (12–121.5) | 0.0912 |
| DAS28-CRP (IQR) | 4.96 (4.11–5.80) | 4.47 (3.58–5.39) | 0.0237* |
| DAS28-ESR (IQR) | 5.46 (4.61–6.40) | 5.13 (4.25–6.07) | 0.0392* |
| Body weight (kg) (IQR) | 52.2 (46.3–58.3) | 52.0 (46.2–59.7) | 0.7608 |
| No. of previous bDMARD/tsDMARD uses (IQR) | 1 (1–2) | 1 (1–2) | 0.808 |
| Prophylaxis with an antitubercular agent | 64/83 (77.1%) | 131/192 (68.2%) | 0.1501 |
| Prophylaxis with TMP-SMX | 3/83 (3.61%) | 3/192 (1.56%) | 0.3705 |
| No. of PSL ≥5mg users | 33/83 (39.8%) | 82/192 (42.7%) | 0.7614 |

| | | | |
|---|---|---|---|
| MTX use | 39/83 (47.0%) | 124/192 (64.6%) | 0.0230* |
| Diabetes mellitus (%) | 16/83 (19.3%) | 17/192 (8.85%) | 0.0245* |
| Initiation of bDMARD/tsDMARD before 2010 | 33/83 (39.8%) | 90/192 (46.9%) | 0.2931 |

DAS28: Disease Activity Score including 28-joint count, CRP: C-reactive protein, bDMARD: biological disease-modifying antirheumatic drug, tsDMARDs: targeted synthetic disease-modifying antirheumatic drug, MTX: methotrexate, TMP-SMX: trimethoprim-sulfamethoxazole. Values are median (interquartile: IQR). The *p*-values were determined using the nonparametric Wilcoxon signed rank test and Fisher's exact test.
